# Stemness Subtypes and Scoring System Predict Prognosis and Efficacy of Immunotherapy in Soft Tissue Sarcoma

**DOI:** 10.3389/fimmu.2022.796606

**Published:** 2022-04-07

**Authors:** Hui-Yun Gu, Wen-Qiang Qu, Hai-Heng Peng, Yi-Feng Yu, Zhe-Zhen Jiang, Bai-Wen Qi, Ai-Xi Yu

**Affiliations:** Department of Orthopedics Trauma and Microsurgery, Zhongnan Hospital of Wuhan University, Wuhan, China

**Keywords:** stemness subtypes, soft tissue sarcoma, prognosis, immunotherapy, multi-omic study

## Abstract

Tumor stemness has been reported to play important roles in cancers. However, a comprehensive analysis of tumor stemness remains to be performed to investigate the specific mechanisms and practical values of stemness in soft tissue sarcomas (STS). Here, we applied machine learning to muti-omic data of patients from TCGA-SARC and GSE21050 cohorts to reveal important roles of stemness in STS. We demonstrated limited roles of existing mRNAsi in clinical application. Therefore, based on stemness-related signatures (SRSs), we identified three stemness subtypes with distinct stemness, immune, and metabolic characteristics using consensus clustering. The low-stemness subtype had better prognosis, activated innate and adaptive immunity (e.g., infiltrating B, DC, Th1, CD8+ T, activated NK, gamma delta T cells, and M1 macrophages), more enrichment of metabolic pathways, more sites with higher methylation level, higher gene mutations, CNA burdens, and immunogenicity indicators. Furthermore, the 16 SRS-based stemness prognostic index (SPi) was developed, and we found that low-SPi patients with low stemness had better prognosis and other characteristics similar to those in the low-stemness subtype. Besides, low-stemness subtype and low-SPi patients could benefit from immunotherapy. The predictive value of SPi in immunotherapy was more accurate after the addition of MSI into SPi. MSI^low^SPi^low^ patients might be more sensitive to immunotherapy. In conclusion, we highlighted mechanisms and practical values of the stemness in STS. We also recommended the combination of MSI and SPi which is a promising tool to predict prognosis and achieve precise treatments of immunotherapy in STS.

## Introduction

Soft tissue sarcomas (STS) are a sort of mesenchymal malignancies which are considered rare and invasive, accounting for less than 1% of malignant tumors (1). Despite lower incidence, STS can be involved in patients with different ages and occur in any anatomical site (2). STS are composed of at least 100 different histological subtypes, each of which has its own biological characteristics and prognostic outcomes (3). Furthermore, patients receiving treatments also had a high probability of recurrence (4). Therefore, the complexity and high heterogeneity present great challenges in the management of STS. Currently, limited efficacy was received in STS, and more effective approaches were required to ameliorate the present predicament. Immunotherapy with a breakthrough in management of tumors might also be suitable for STS. However, individual responses to immunotherapy vary greatly, leading to treatment failure in STS (5). Therefore, it is imperative to comprehensively study mechanisms leading to different responses to immunotherapy and develop tools to identify patients who are more sensitive to immunotherapy.

Tumor stemness plays important roles in tumors and could assess characteristics of the cancer stem cells (CSCs) (6). CSCs, a small subgroup of cancer cells found in primitive tumor cells, possess stem cell-like characteristics, such as self-renewal and differentiation (7). The stem cell-like characteristics also lead to the generation of tumor cells from CSCs (8) and guarantee the close relationships of CSCs with tumor growth, metastasis, recurrence, and drug resistance. In STS, various studies identified CSCs (9–11), with the ability to produce different cell types and facilitate the heterogeneity (12), which are responsible for the development of resistance to various oncologic treatments, such as chemotherapy and radiotherapy (13). Besides, aberrant stemness gene expression and stemness-related biological pathways (Hedgehog pathway, Hippo pathway, and Notch pathway) were also found in STS (14). It has also been shown that the poor prognosis of patients was associated with the high expression of molecular characteristics related to tumor stem cells (15). Despite the harmful roles of stemness or CSCs in tumors, the reversibility and plasticity of SCSs provide potentials in anticancer therapy. It is well accepted that multiple cells including relatively differentiated tumor cells, CSCs, infiltrating immune cells, tumor-associated fibroblasts, endothelial cells, and other cell types participate in the formation of tumors (6). The common microenvironment may contribute to the close communication and relationships of CSCs with immune cells. In acute myeloid leukemia, compared with tumor cells, CSCs with the absence of NKG2D ligands were more likely to escape immune killing (16). A study focusing on bladder cancer demonstrated that immune checkpoint molecule PD-L1 was significantly upregulated in patients with high stemness (17). This molecule inhibits the proliferation and differentiation of T lymphocytes and promotes the differentiation of regulatory T cells, thereby suppressing the immune response (18). Similar results were found in prostate cancer (19). In addition, tumor stemness was also associated with poor immune response in STS (20, 21). Therefore, interfering with disordered CSCs or stemness may remodel the immune microenvironment to promote the application of immunotherapy in STS. However, compared with other tumors, little is known about the detailed relationship between stemness or CSCs and the immune-infiltrating environment, and the mechanisms and practical values of stemness in STS, which needs to be fully analyzed and understood.

In this study, based on tumor stemness-related genes, we obtained different stemness subtypes using machine learning algorithm. Muti-omic data was used to reveal different characteristics of stemness subtypes. We also demonstrated important roles of stemness in STS. Finally, a scoring system was developed to guide the clinical application of stemness for prediction of prognosis and responses of patients to immunotherapy in STS. Our study provides new insights into tumor stemness and helps to improve clinical management of STS.

## Methods and Materials

### Data Collection and Preprocessing

STS samples were searched through The Cancer Genome Atlas Program (TCGA) and Gene Expression Omnibus (GEO) databases. Normalized gene expression data (FPKM values) of TCGA-SARC, obtained using the RNA-sequencing method, was downloaded from the UCSC (University of California, Santa Cruz) Xena browser (https://gdc.xenahubs.net). However, gene expression data of GSE21050 (22) were generated from the microarray, different from the RNA-sequencing method. We downloaded the gene expression data of GSE21050 which had been normalized using the GCRMA (GC-Robust Multi-Array Analysis) algorithm by the previous study (22). We performed the following procedures to make them more comparable between TCGA-SARC and GSE21050 cohorts. Firstly, we excluded samples with incomplete survival information in two cohorts. Then, FPKM (fragments per kilobase of transcript per million mapped reads) values of TCGA-SARC were transformed into TPM (transcripts per kilobase million) values which were more similar to those of GSE21050 (23). Furthermore, normalization and removal of batch effects between two cohorts were performed using the “sva” package (24) of R 4.0.3 software. Besides, DNA methylation data (450K), somatic mutation data, and copy number alteration (CNA) data were also downloaded from the UCSC Xena browser.

### Identification of Stemness-Related Signatures for STS

Across published literatures, Malta et al. (6) provided two dependent stemness indices (mDNAsi and mRNAsi), which work well in assessment of cancer stemness. mRNAsi calculated based on gene expression features rather than mDNAsi generated based on epigenetic features was downloaded to identify stemness-related signatures (SRSs) by application of weighted gene co-expression network analysis (WGCNA) to gene expression data. In Malta’s work, a predictive model using one-class logistic regression was applied to determine stem cell signatures based on mRNA expression from the pluripotent stem cell samples. Then, mRNAsi was calculated as Spearman correlations between the model’s weight vector and the new sample’s signature expression (6). In this study, mRNAsi was chosen because gene expression data are more common and easier to obtain than epigenetic data. WGCNA, a systems biology approach, with unique superiority in processing high-dimensional and large-scale data, is increasingly used to find genes most associated with phenotypic traits (25, 26). The identification of SRSs for STS relied on the “WGCNA” package in R 4.0.3 software, along with its official manual (27). Briefly, 3,622 genes associated with stem cells from 26 human stem cell gene sets were collected from StemChecker (http://stemchecker.sysbiolab.eu/) (28). The gene expression matrix containing 3,622 genes of TCGA-SARC was used to calculate co-expression similarity. Based on soft-thresholding(β), co-expression similarity was transformed into the adjacency matrix. Then, hierarchical clustering and dynamic tree cut methods were used to identify gene modules. To determine interesting gene modules, we related different gene modules to the phenotypic trait (mRNAsi). In this study, we defined interesting gene modules as gene modules with the highest correlation coefficient with mRNAsi. Finally, genes in interesting gene modules were regarded as SRSs.

### Molecular Subtypes Based on Prognostic SRSs

SRSs were extracted from interesting gene modules and then were subjected to univariate Cox regression analysis. To reveal the role of prognostic SRSs (p < 0.01), we tried to explore potential tumor stemness status in STS. The “ConsensusClusterPlus” package (29) was used to perform the consensus clustering (K-means) algorithm, which was repeated 50 times to obtain reliable stemness subtypes for STS. Regarding the determination of the optimal clustering number (K value), we mainly referred to the consensus matrix and empirical cumulative distribution function plots. Subsequently, Kaplan–Meier (K–M) survival analysis was further used to evaluate the accuracy and practicability of stemness subtypes in STS. In this study, K–M survival curves were plotted and survival differences were determined using “survdiff” function based on “survminer” packages.

### Biological Differences in Different Stemness Subtypes

To investigate biological differences in different stemness subtypes, we characterized the immune microenvironment through multiple methods. Firstly, immune scores and stromal scores were calculated based on the “estimate” package, which works dependent on an algorithm, named as Estimation of STromal and Immune cells in MAlignant Tumours using Expression data (ESTIMATE) (30). Immune scores and stromal scores provided a preliminary assessment of the immune microenvironment for STS. Secondly, the “immunedeconv” package (31) containing multiple algorithms (TIMER, xCell, MCP-counter, CIBERSORT, EPIC, quanTIseq, and IPS) was applied to quantify the abundance of different immune cells in STS samples. Thirdly, 29 immune gene sets reflective of innate and adaptive immunity were searched from previous studies (32, 33) and then were subjected to single-sample gene set enrichment analysis (ssGSEA) to reveal immune status in different stemness subtypes. In addition to the characterization of the immune microenvironment, we preformed gene set variation analysis(GSVA) (34) to further score different biological pathways based on background gene sets (c2.cp.kegg.v7.4.symbols) from MSigDB (Molecular Signatures Database v7.4) in the GSEA official website (http://www.gsea-msigdb.org/gsea/msigdb/index.jsp). According to scores of different biological pathways, the biological status of stemness subtypes was fully evaluated in STS samples.

### The Identification and Functional Annotation of Differentially Expressed Genes

To further understand the effect of tumor stemness on biological status in STS, differentially expressed genes (DEGs) among different stemness subtypes were identified using the “limma” package (35), based on two requirements: |logFC(fold change)| >2 and FDR (false discovery rate) <0.05. Gene Ontology (GO) and Kyoto Encyclopedia of Genes and Genomes (KEGG) pathway analyses were well-accepted procedures to annotate DEGs. Then, the “clusterProfiler” package (36) was used to perform functional annotation for DEGs to investigate the involved biological processes and pathways. Background gene sets of functional annotation were sourced from the GO or KEGG database. Enriched terms were visualized according to significant criteria (p and q values less than 0.05).

### Somatic Mutation and CNA Among Stemness Subtypes

After downloading the somatic mutation data (MuTect2 Variant Aggregation and Masking) from TCGA-SARC, the “maftools” package was used to calculate the frequency of gene mutation in specific patients. We tried to find potential gene mutation driver cancer stemness in STS through the identification of differential gene mutations among stemness subtypes. Somatic mutation data were also applied to obtain tumor mutation burden (TMB) according to previous studies (37, 38). Furthermore, we indirectly quantified frequencies of gene mutation for patients among stemness subtypes using TMB.

To reveal the potential impact of CNA on stemness in STS, we requested CNA data from the UCSC Xena browser. The comparison of CNA between STS samples with the highest and lowest stemness was performed using the chi-square test (p < 0.001). Genes with significant CNA were visualized with the “RCircos” package (39) and subjected to functional annotation with GO and KEGG analyses.

### DNA Methylation Analysis and Identification of Stemness-Related Methylation-Driven Genes

We obtained DNA methylation data (450K) of TCGA-SARC to perform the following procedures: 1) Only DNA methylation data of STS samples included in this study were reserved for subsequent analyses. 2) Sites with deletion values greater than 70% and located in the X and Y chromosomes were deleted. 3) We identified sites surrounding the transcription start sites (TSS) (-200 to -1,500 bp) (40), which were mostly located in the promoter region and had a negative regulation of gene expression. To find stemness-related differential methylation probes, we performed differential analysis between STS samples with the highest and lowest stemness, based on |logFC| >0.25 and adjusted p value <0.05. In addition, the “MethyMix” package (41) was used to find stemness-related methylation-driven genes, which were required to meet three criteria: 1) They should be DEGs (|logFC| >1.5 and FDR <0.05); 2) they should be differentially methylated genes (|logFC| >0.5 and p <0.05); and 3) the correlation coefficient of methylation level and gene expression should be less than -0.3.

### Generation of Stemness Prognostic Index

Considering that quantitation of stemness subtypes would contribute to clinical application, we developed a set of scoring tool based on SRSs. Common genes between SRSs and DEGs were determined for the calculation of the stemness prognostic index (SPi) using least absolute shrinkage and selection operator (LASSO) regression analysis, performed using the “glmnet” package. Through 1,000 times cross-validation, reliable and optimal genes were remained to generate SPi. Then, the optimal cutoff was determined to achieve perfect prognostic stratification for STS patients. Based on the optimal cutoff, patients with SPi more than the optimal cutoff were assigned to the high-SPi group. Similarly, low SPi was also generated. K–M survival analysis was used to compare survival differences between the high- and low-SPi groups. Furthermore, the receiver operating characteristic (ROC) curve was used to assess the prognostic performance of SPi in STS. It is noted that SPi was trained in TCGA-SARC cohort and tested in the GSE21050 and overall cohorts (TCGA-SARC + GSE21050).

### Multi-Omic Analysis for SPi

To better reveal the characteristics and accuracy of SPi in STS, we performed series analyses: 1) clinical characteristics; 2) immune infiltration; 3) biological processes based on GSEA analysis; 4) somatic mutation; and 5) tumor immunogenicity analysis. We performed GSEA analysis to evaluate different biological processes between patients with high and low SPi. Furthermore, tumor immunogenicity indicators including TMB, neoantigen burden (defined as the total predicted neoantigen count), DNA damage including homologous recombination deficiency (HRD), loss of heterozygosity (LOH; number of segments with LOH events, and fraction of bases with LOH events), and intratumor heterogeneity (ITH) were compared between high- and low-SPi patients. Except for TMB, all other immunogenicity indicators were sourced from a previous study (42).

### Prediction of Response to Immunotherapy

We used the following methods to ensure the accuracy of stemness in the prediction of response to immunotherapy. Firstly, T-cell inflammatory scores (TIS) were calculated by GSVA analysis. Patients with a higher TIS were more likely to benefit from immune checkpoint inhibitors (43). Secondly, tumor immune dysfunction and exclusion (TIDE) scores superior to PD-1 or TMB in the prediction of response to immunotherapy (44) were obtained through an official website (http://tide.dfci.harvard.edu/). Opposite to TIS, lower TIDE scores indicated more response to immunotherapy. Besides, we assessed the predictive value of SPi in response of patients to immunotherapy using a melanoma cohort containing 47 patients treated with CTLA-4 blockade and PD-1 blockade (45). The subclass mapping (SubMap) method was utilized, and the results were visualized by the “complexHeatmap” package (46).

## Results

### The Role of mRNAsi in STS

**Figure 1:**
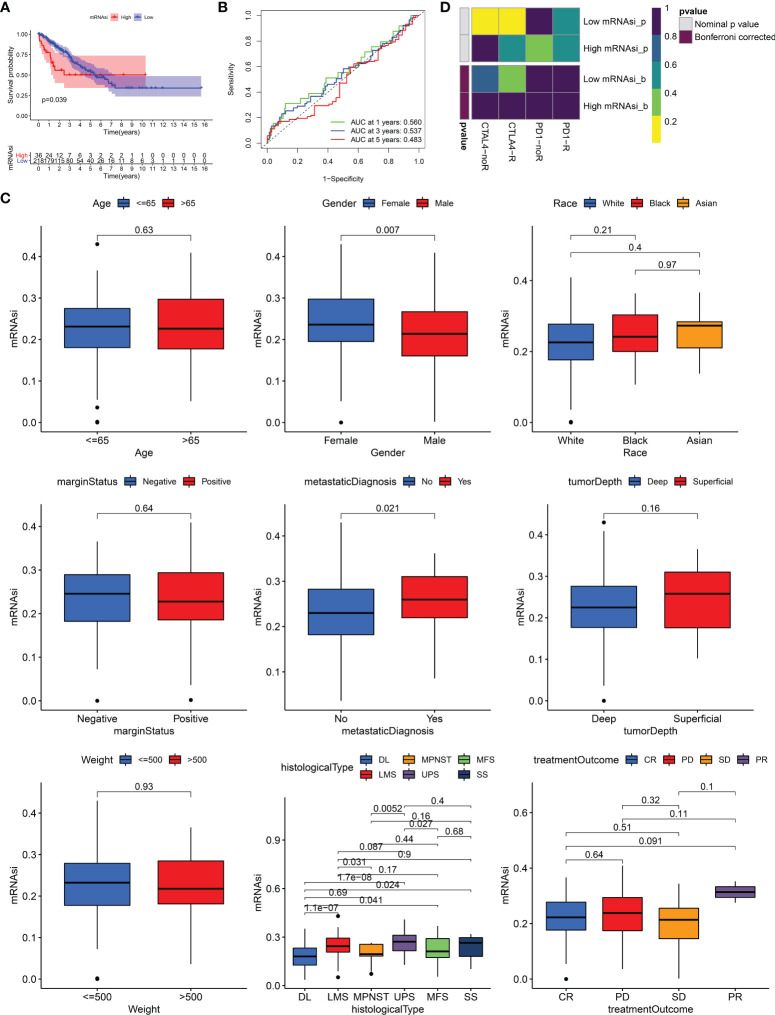
The role of mRNAsi in STS. **(A)** K–M survival analysis of patients with high and low mRNAsi. **(B)** The receiver operating characteristic (ROC) curve of mRNAsi in predicting survival of STS. **(C)** The differences of mRNAsi in patients with different characteristics. DL, dedifferentiated liposarcoma; MPNST, malignant peripheral nerve sheath tumors; MFS, myxofibrosarcoma; LMS, leiomyosarcoma; UPS, undifferentiated pleomorphic sarcoma; SS, synovial sarcoma; CR, complete response; PD, progressive disease; SD, stable disease; PR, partial response. **(D)** The response of patients with high and low mRNAsi to PD1 and CTLA4 inhibitors (Benjamini and Hochberg corrected p > 0.05).

259 STS patients from TCGA-SARC cohort and 309 from the GSE21050 cohort were included in this study. Due to clinical data missing in the GSE21050 cohort, we only revealed the role of mRNAsi in TCGA-SARC cohort. Firstly, survival analysis showed that mRNAsi could not perform well in the risk stratification of STS patients with different prognoses, although a significant p value was obtained (p = 0.039, **Figure 1A**). The ROC curve also demonstrated the unsatisfactory prognostic value of mRNAsi in STS (AUC = 0.560, 0.537, 0.483 at 1, 3, and 5 years, respectively, **Figure 1B**). Then, the clinical characteristics of mRNAsi were investigated. **Figure 1C** shows that female patients (p = 0.007) and metastatic patients (p = 0.021) had higher mRNAsi. In terms of six histological types of STS, higher mRNAsi was observed in patients diagnosed with leiomyosarcoma (LMS) and undifferentiated pleomorphic sarcoma (UPS); however, lower mRNAsi was seen in dedifferentiated liposarcoma (DL) (**Figure 1C**). Moreover, patients with different responses to treatments had similar mRNAsi (p > 0.05). To explore the predictive value of mRNAsi in immunotherapy, we performed SubMap analysis, and results are displayed in **Figure 1D**. SubMap analysis revealed that mRNAsi had no guiding significance for the use of CTLA4 blockade or PD-1 blockade in STS (p > 0.05).

### Identification of Three Stemness Subtypes Based on Prognostic SRSs

**Figure 2:**
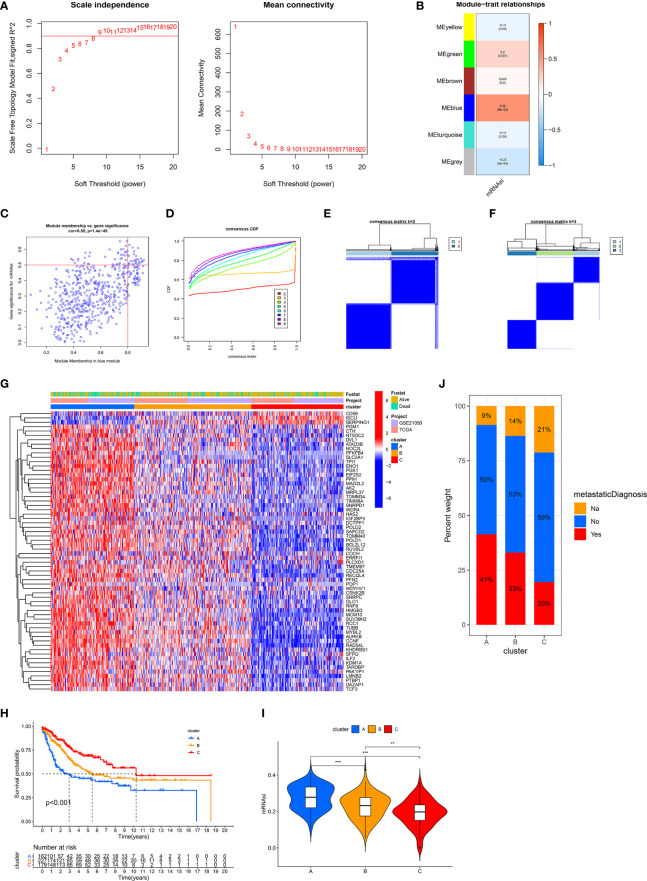
Identification of three stemness subtypes based on prognostic SRSs. **(A–C)** Identification of SRSs based on WGCNA. **(A)** Selection of the optimal soft threshold power, β (optimal β = 8, R^2^ = 0.9). **(B)** Heatmap of the module–trait relationship. Six modules were identified and related to clinical traits. The color depth of each cell represents the correlation between the module and mRNAsi, and the numbers in the cell represent the correlation coefficients and p value, respectively. **(C)** Identification of genes with high significance and module membership in the mRNAsi-related blue module. **(D–F)** Consensus clustering (K-means) algorithm was performed for overall patients. **(C)** CDF plot. The flatter the middle part of the curve, the better the clustering effect. **(E, F)** Consensus matrix plots. K = 3 was determined as optimal clustering number. **(G)** Heatmap of the gene expression of prognostic SRSs among three stemness subtypes (clusters A, B, and C). **(H)** K–M survival analysis in clusters A, B, and C. **(I)** The differences of mRNAsi among clusters A, B, and C. **(J)** The percentage of metastatic and non-metastatic patients. Na: data were not available. ***p < 0.001; **0.001 < p < 0.01.

Due to the limited roles of mRNAsi in prognostic prediction and the use of immunotherapy in STS, new stemness-related tools such as molecular subtypes or a scoring system were required. We carried out WGCNA to identify SRSs to develop new stemness-related tools. 3,622 genes were prepared for WGCNA. Then, β = 8 was used to transform co-expression similarity into the adjacency matrix (**Figure 2A**), and six gene modules were obtained through hierarchical clustering and dynamic tree cut methods. We found that the blue module, containing 560 genes, had the highest positive correlation with mRNAsi (cor = 0.56, p = 6e-22, **Figure 2B**). Furthermore, a high correlation of the module membership with gene significance is observed in **Figure 2C** (cor = 0.55, p = 1.4e-45). In addition, univariate Cox regression analysis identified 64 prognostic SRSs (**Table S1**).

Based on prognostic SRSs, consensus clustering analysis provided different clustering numbers (k = 2–9, **Figure 2D**). “Cleanest” clustering was observed when k = 2 or 3 (**Figures 2E, F**). We classified the overall cohort (568 STS samples) into three subtypes (clusters A, B, and C) because more clustering numbers could contribute to understanding characteristics and the application of stemness subtypes. Then, we investigated some characteristics of three subtypes. Three stemness subtypes had distinct SRS gene expression patterns and survival rates (**Figures 2G, H**). Survival analysis showed that cluster C (179 patients) had the best prognosis, followed by cluster B (227 patients), and cluster A (162 patients) had the worst prognosis. Besides, the stemness of three stemness subtypes was quantified by mRNAsi, and we found that cluster A had the highest stemness, followed by clusters B and C (**Figure 2I**). In addition, the close relationship between stemness and metastasis of cancer is demonstrated in **Figure 2J**. Cluster A (high stemness) tended to develop tumor metastasis with a percentage of 41%, twice that in cluster C (low stemness). The accuracy of stemness subtypes was also evaluated and validated in TCGA-SARC (**Figure S1**) and GSE21050 (**Figure S2**) cohorts.

### Distinct Biological Differences in Stemness Subtypes

**Figure 3:**
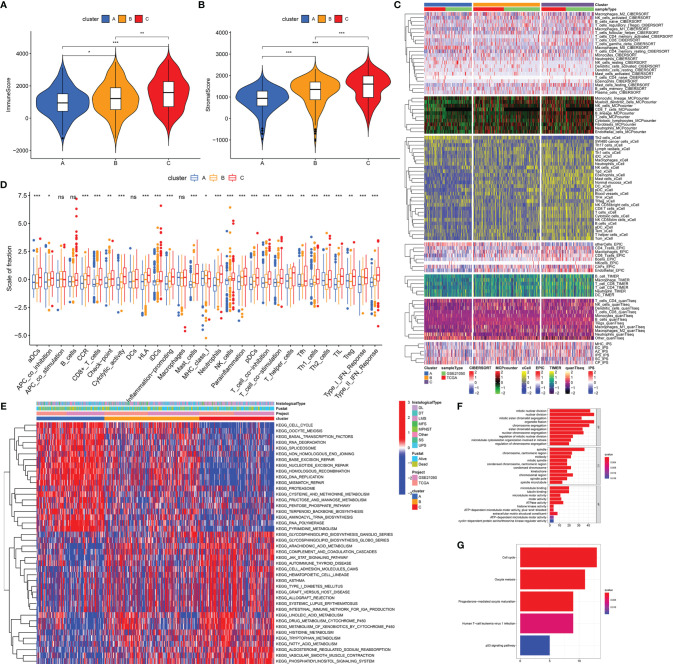
Distinct biological differences in stemness subtypes. The differences of immune **(A)** and stromal **(B)** scores among three subtypes (clusters A, B, and C) in the overall cohort. **(C)** The heatmap of immune infiltration (calculated by seven methods) among three subtypes. **(D)** The differences of enrichment scores of 29 immune gene sets reflective of innate and adaptive immunity among three subtypes. **(E)** The heatmap of differences of GSVA scores of KEGG pathways. GO **(F)** and KEGG **(G)** analyses of DEGs from three stemness subtypes. ***p < 0.001; **0.001 < p < 0.01; *0.01 < p < 0.05; ns (not significant), p > 0.05.

We performed biological analyses to further clarify the differences of three stemness subtypes. Estimation of immune and stromal components revealed that Cluster C was characterized by higher immune scores, compared with clusters A and B (p < 0.05, **Figure 3A**). Interestingly, higher stromal components were also seen in cluster C (p < 0.05, **Figure 3B**). Detailed immune and stromal components estimated by seven methods also demonstrated high immune and stromal status in cluster C and are clarified in **Figure 3C**. Compared with cluster A characterized by worst prognosis, cluster C, characterized by best prognosis, possessed more B, DC, Th1, CD8+ T, activated NK, gamma delta T cells, and M1 macrophages and normal mucosa cells. However, cluster A had more Th2, M0, Treg, and SW480 cancer cells. Besides, scores from 29 immune gene sets based on the ssGSEA method also supported these findings. **Figure 3D** shows that innate and adaptive immunities were both activated in cluster C patients. Similar results obtained from TCGA-SARC (**Figure S3**) and GSE21050 (**Figure S4**) cohorts further witnessed the accuracy of the immune microenvironment in the specific stemness subtype. In addition to the immune microenvironment, important biological pathways were studied to illustrate the biological differences in stemness subtypes. It is noted that patients with the lowest stemness (cluster C) possessed higher tumor immune, metabolic levels (drug, histidine, tryptophan, fatty acid metabolism), an elevated phosphatidylinositol signaling system, and vascular smooth muscle contraction (**Figure 3E**). Instead, lots of pathways associated with cell cycle, cell division, and RNA metabolism were activated in cluster A with the highest stemness. We obtained 150 DEGs from different stemness subtypes (|logFC| >2 and FDR <0.05, **Table S2**). Results of GO and KEGG further revealed the biological differences among different stemness subtypes. **Figures 3F, G** show that 150 DEGs were involved in the cell cycle, oocyte meiosis, progesterone-mediated oocyte maturation, human T-cell leukemia virus 1 infection, and p53 signaling pathway (q value <0.05), consistent with findings from GSVA analysis (**Figure 3E**).

### High Stemness Was Associated With More Somatic Mutation and CNA

**Figure 4:**
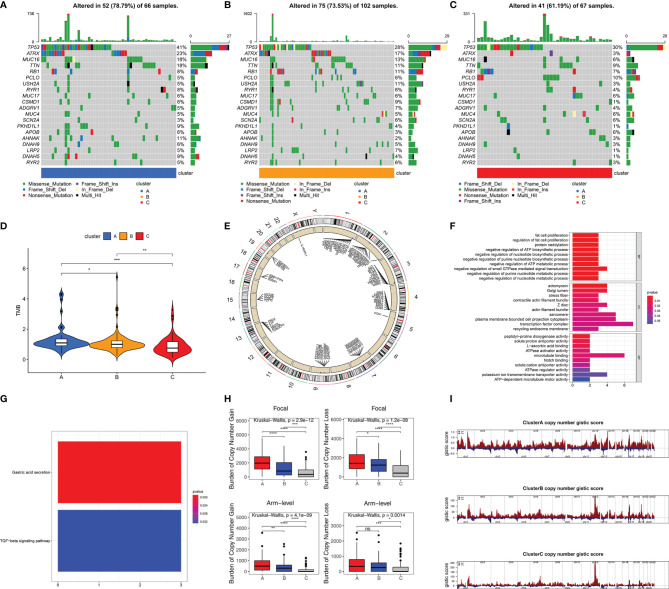
High stemness was associated with more somatic mutation and CNA. Visualization of gene mutations in cluster A **(A)**, cluster B **(B)**, and cluster C **(C)**. **(D)** The difference of TMB in three stemness subtypes. TMB value was subjected to the transformation of log2(X+1). **(E)** The visualization of genes with significant CNAs associated with stemness in highest stemness (cluster A) and lowest stemness (cluster C). GO **(F)** and KEGG **(G)** analyses of genes with significant CNAs associated with stemness. **(H)** The differences of burden of copy number gain and loss among three stemness subtypes. **(I)** The visualization of copy number gistic scores among three stemness subtypes. ****p < 0.0001; ***p < 0.001; **0.001 < p < 0.01; *0.01 < p < 0.05; ns (not significant), p > 0.05.

Somatic mutation data from 235 samples were obtained to investigate differences of gene mutation among stemness subtypes. 78.79% (52/66), 73.53% (75/102), and 61.19% (41/67) samples with gene mutations were observed in clusters A (with highest stemness), B (with intermediate stemness), and C (with lowest stemness), respectively (**Figures 4A–C**). Specific gene mutations are also visualized in **Figures 4A–C**. We used TMB to further demonstrate that high stemness was associated with more somatic mutation (TMB: cluster A > B > C, p < 0.05, **Figure 4D**). Next, we explored CNAs associated with stemness, which were illustrated by the identification of differences of CNAs between samples with highest stemness (cluster A) and lowest stemness (cluster C). Significant CNAs were observed in 184 genes (p < 0.0001, **Figure 4E**). Functional annotation revealed that these genes played vital roles in fat cell proliferation, notch binding, regulation of nucleotide metabolic process, and TGF-beta signaling pathway (**Figures 4F, G**). We found that high stemness was also associated with more CNAs (**Figures 4H, I**). Patients with highest stemness (cluster A) had the highest copy number burden (p < 0.05, **Figure 4H**). Furthermore, compared with cluster C, cluster A had obviously higher gistic scores across 22 chromosomes, especially in chr 1–4, 7, 9, 13, 15, 17, and 19 (**Figures 4E, I**).

### DNA Methylation Analysis and Identification of Stemness-Related Methylation-Driven Genes

**Figure 5:**
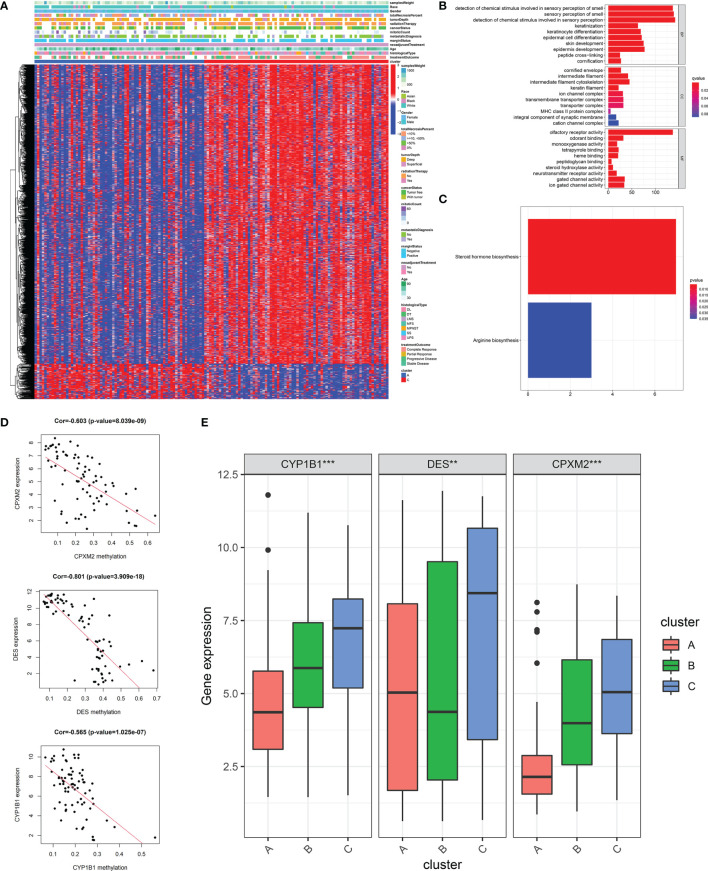
DNA methylation analysis and the identification of stemness-related methylation-driven genes. **(A)**The heatmap of differential methylation sites between highest stemness (cluster A) and lowest stemness (cluster C). GO **(B)** and KEGG **(C)** analyses of genes with differential methylation sites. **(D)** The correlations of gene expression of three stemness-related methylation-driven genes with corresponding value of gene methylation. **(E)** The differences of stemness-related methylation-driven genes among three stemness subtypes. ***p < 0.001; **0.001 < p < 0.01.

To explore the impact of DNA methylation on stemness, we performed differential analysis and identified 2,054 DNA methylation sites between patients with highest (cluster A) and lowest (cluster C) stemness (**Table S3**). More sites with a higher methylation level were found in the lowest stemness group (**Figure 5A**). After mapping to genes, functions of differential methylation sites were uncovered by GO and KEGG analyses. These sites were mainly involved in skin development, epidermis development, arginine biosynthesis, and steroid hormone biosynthesis (**Figures 5B, C**). We further investigated stemness-related methylation-driven genes using the “MethyMix” package. Three genes (CPXM2, CYP1B1, and DES) with significantly negative correlations between gene expression and methylation level were identified as stemness-related methylation-driven genes (CPXM2: cor = -0.603, p value = 8.039e-09; CYP1B1: cor = -0.565, p value = 1.025e-07; DES: cor = -0.801, p value = 3.909e-18, **Figure 5D**). Three genes were also validated in clusters A, B, and C with different stemness (**Figure 5E**).

### Clinical and Multi-Omic Characteristics of SPi

**Figure 6:**
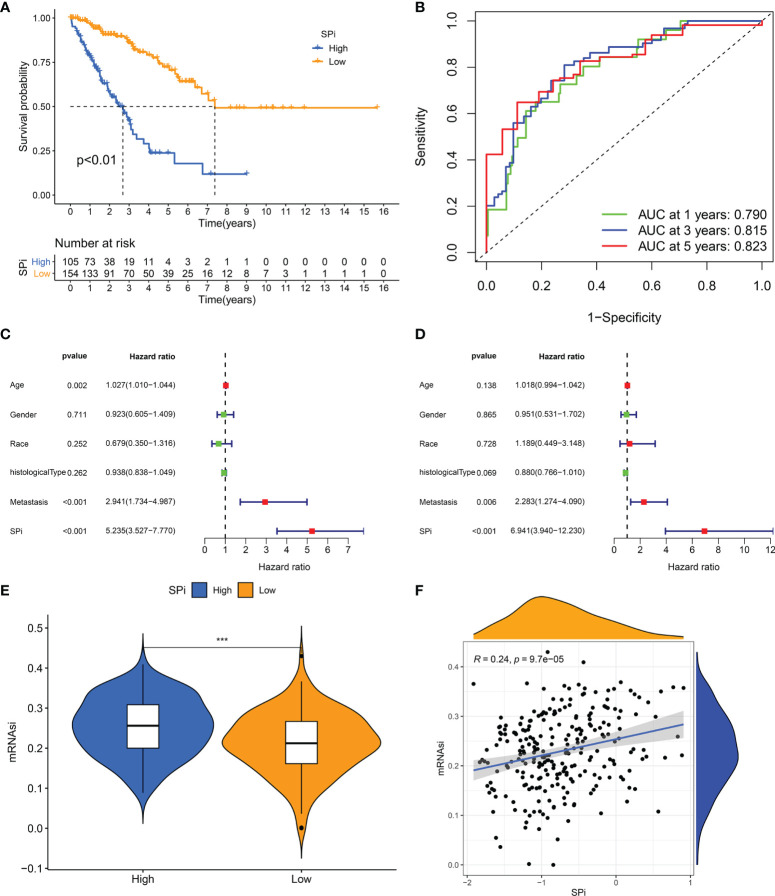
The prognostic role and stemness characteristic of SPi in STS from TCGA cohort. **(A)** K–M survival analysis of patients with high and low SPi. **(B)** ROC curve of SPi at 1, 3, and 5 years. Forest plot of the univariate **(C)** and multivariate **(D)** analyses for the clinical factors and SPi in STS. **(E)** The difference of mRNAsi between high-SPi patients and low-SPi patients. **(F)** The correlation of SPi with mRNAsi. ***p < 0.001.

A quantitative tool was determined to contribute to clinical application of SRSs in STS. 57 genes derived from the intersection of SRSs and DEGs were subjected to LASSO analysis, and 16 optimal genes were used to construct a SRS-related model, termed as SPi (SPi = -0.011*S100A2 + 0.396*RAD54L + 0.160*AURKB + 0.104*TRIP13 + 0.089* MAD2L1 + 0.030* KIF15-0.048* CKS2-0.278*CEP55 + 0.027*PBK + 0.058*TK1-0.050*PRC1-0.277*OIP5-0.079*UBE2T + 0.134* SLC2A1-0.145*SERPING1-0.061*IGF1). We performed a series of analyses to reveal characteristics of SPi in TCGA-SARC cohort, because of its relative complete clinical and multi-omic data. We found that SPi was associated with survival. According to the optimal cutoff of SPi, patients were divided into two groups. Low-SPi patients had better prognosis (**Figure 6A**), which was also validated in **Figures S5A, B**. Compared with mRNAsi, SPi had excellent prognostic value (**Figure 1B** and **Figure 6B**). **Figures 6C, D** indicate that SPi was the independent prognostic factor for STS. Besides, the difference of stemness between high-SPi and low-SPi patients was assessed using mRNAsi in **Figure 6E**. High-SPi patients were characterized by high stemness (mRNAsi), which was consistent with a positive correlation between SPi and mRNAsi (cor = 0.24, p = 9.7e-05, **Figure 6F**). Then, we assessed clinical characteristics of SPi (**Figure S5C**). Patients aged >65 and diagnosed with UPS had higher SPi. High-SPi patients with poor prognosis tended to develop metastasis (p = 0.014). Low-SPi patients were more likely to respond to treatments.

**Figure 7:**
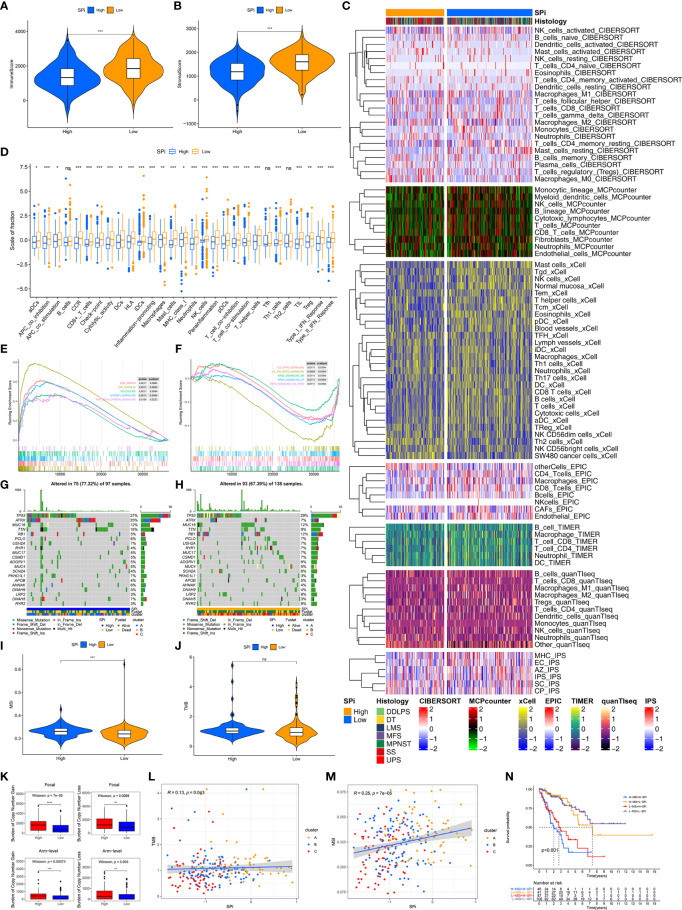
Multi-omic characteristics of SPi. The differences of immune **(A)** and stromal **(B)** scores between patients with high and low SPi. **(C)** The landscape of immune infiltration in patients with high and low SPi. **(D)** The differences of enrichment scores of 29 immune gene sets reflective of innate and adaptive immunity in patients with high and low SPi. GSEA of patients with high SPi **(E)** and low SPi **(F)**. **(G)** Visualization of gene mutations in patients with high **(G)** and low SPi **(H)**. The differences of MSI **(I)** and TMB **(J)** between patients with high and low SPi. TMB value was subjected to the transformation of log2(X+1). **(K)** The differences of burden of copy number gain and loss between patients with high and low SPi. The correlation of TMB **(L)** and MSI **(M)** with SPi. **(N)** Survival analyses for patients with high MSI+ high SPi, high MSI +low SPi, low MSI + high SPi, and low MSI + low SPi. ***p < 0.001; **0.001 < p < 0.01; *0.01 < p < 0.05; ns (not significant), p > 0.05.

Immune characteristics of SPi were also investigated as shown in **Figure 7**. Low-SPi patients had higher immune, stromal scores and more infiltration of immune cells (**Figures 7A–C**). Furthermore, low-SPi patients also had activated innate and adaptive immunity, assessed using the ssGSEA method (**Figure 7D**). **Figure 7E** shows that DNA repair, E2F targets, glycolysis, mtorc1 signaling, and WNT beta catenin signaling pathways were enriched in patients with high SPi. Instead, immune pathways, KRAS signaling, and TNFA signaling *via* NF-KB were activated in low-SPi patients (**Figure 7F**).

In addition to transcriptome analysis, we also investigated differences of gene mutations between patients with high and low SPi. As we expected, more proportions of gene mutation were found in patients with high SPi (**Figures 7G, H**), and high SPi was associated with high MSI and CNA burden (**Figures 7I, K**). However, there was no significant difference of TMB between high-SPi and low-SPi patients (**Figure 7J**). Besides, significant correlations between SPi and TMB (cor = 0.13, p = 0.043) or MSI (cor = 0.25, p = 7e-05) were as shown in **Figures 7L, M**. Based on these findings, considering the closer relationship between SPi and MSI, we introduced MSI into SPi to achieve an accurate classification of patients with different prognoses. **Figure 7N** shows the combination of SPi and MSI; patients with STS were classified into four groups with markedly different prognoses (p < 0.001). Furthermore, high SPi was also associated with high immunogenic biomarkers including AS, HRD, ITH, and LOH, but not neoantigen burden (**Figure S6**).

### Patients With Low Stemness Could Benefit From Immunotherapy

**Figure 8:**
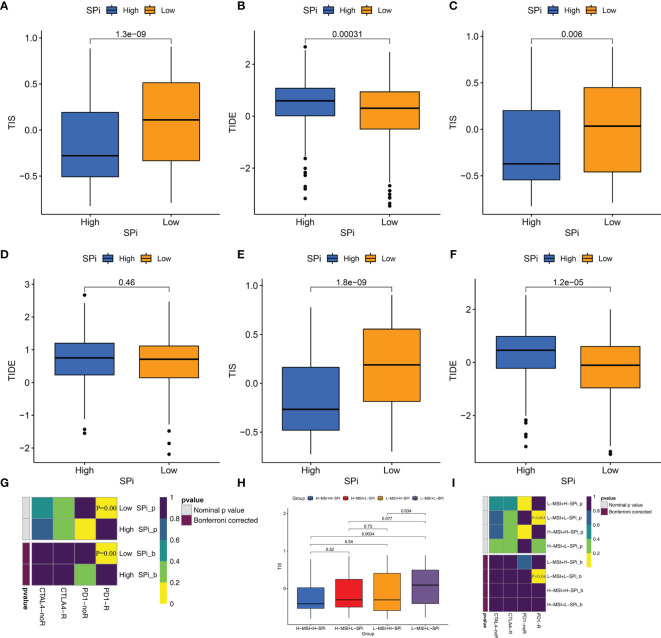
Patients with low stemness could benefit from immunotherapy. The differences of TIS and TIDE between patients with high and low SPi in the overall cohort **(A, B)**, TCGA-SARC cohort **(C, D)** GSE21050 cohort **(E, F)**. **(G)** The response of patients with high and low SPi to PD1 and CTLA4 inhibitors (Benjamini and Hochberg corrected p < 0.05). **(H)** The differences of TIS in patients with high MSI+ high SPi, high MSI +low SPi, low MSI + high SPi, and low MSI + low SPi. **(I)** The response of patients with high MSI+ high SPi, high MSI +low SPi, low MSI + high SPi, and low MSI + low SPi to PD1 and CTLA4 inhibitors (Benjamini and Hochberg corrected p < 0.05).

Whether stemness was associated with response to immunotherapy was investigated in this study. As is shown in **Figures 8** and **S7**, three methods were used to identify patients who respond to immunotherapy. Compared with cluster A, cluster C with low stemness had high TIS (**Figures S7A, C, E**) and low TIDE scores (**Figures S7B, D, F**). Although the difference of TIDE scores between clusters A and C did not reach significance (p = 0.095), **Figure S7D** shows that relatively lower TIDE scores were observed in cluster C. We also performed SubMap analysis to predict immunotherapy in **Figure S7G**. Patients in cluster C were more sensitive to PD-1 blockade. Similar to stemness subtypes, **Figures 8A–G** show that SPi could serve as an indicator to predict the efficacy of immunotherapy. **Figures 8H, I** indicates that MSI (adjusted p = 0.04) could help SPi to identify patients (with low MSI and SPi) more sensitive to immunotherapy.

## Discussion

STS with low incidence received less attention from researchers. However, more complexity and high heterogeneity lead to the dilemma of management of STS. More seriously, little knowledge about STS was obtained. Hence, STS deserves enough attention to contribute to management.

It was well accepted that mRNAsi from a previous study (6) was a great evaluation indicator for tumor stemness. Tumor stemness played important roles in biological behaviors of tumor including metastasis, resistance to treatment, and maintenance of the tumor microenvironment (47), which was consistent with our findings in STS. However, little role of mRNAsi in the prediction of prognosis and immunotherapy was found in STS, which limited the clinical application of mRNAsi. Due to vital roles of stemness in STS, further studies on stemness could promote the clinical management of STS. We introduced stemness into STS to comprehensively study the role of tumor stemness in STS. Based on two well-established cohorts, we identified three stemness subtypes and obtained SPi, which performed well in the prediction of prognosis and response to immunotherapy.

In this study, based on prognostic SRSs, three stemness subtypes with distinct prognosis and biological characteristics were obtained. Patients with high stemness had worse prognosis and were more likely to develop metastases. To further explore mechanisms leading to distinct characteristics in stemness subtypes, we performed multilevel analysis for patients with STS. Immune and stromal components, which could reflect innate and adaptive immune status and were well recognized as prognostic factors for tumors (48), were analyzed for each stemness subtype. We found that different survival differences might be caused by immune infiltration. Specifically, B, DC, Th1, CD8+ T, activated NK, gamma delta T cells, and M1 macrophages with ability to directly or indirectly kill abnormal tumor cells (48–50) might support better prognosis in low-stemness subtypes. However, poor prognosis in high-stemness subtypes was illustrated by low immune infiltration of immune killer cells and more immunosuppressive cells. Besides, higher immune inhibition might also occur in patients with activated immune status. The above findings supported our speculation that immune escape was more likely to occur in STS cells with high stemness, resulting in poor prognosis.

In addition to high immune status, low stemness was also characterized by high metabolic status. Histidine metabolism was enriched in patients with low stemness and better prognosis, consistent with a previous study (51). Close relationships of drug metabolism cytochrome p450, tryptophan and fatty acid metabolism with prognosis, and tumor stemness were also reported in previous studies (52, 53) and found in this study. Instead, patients with high stemness were characterized by higher activity of the cell cycle, cell division, DNA replication, and mismatch repair, which were prone to generate abnormal mutations and result in malignant proliferation of tumors (54, 55). Functional annotation of DEGs from stemness subtypes further supported the above results. Therefore, STS with different stemness might maintain malignant potential by influencing tumor immunity and metabolism.

Due to complexity of the cancers, multi-omics analysis was necessary to better reveal the pathogenesis of STS. Mounting evidence found that gene mutations could cause cell abnormalities and uncontrolled growth, leading to the occurrence and progression of tumors (56). We found that patients with high stemness were more likely to develop genetic mutations, resulting from higher activity of the cell cycle, cell division, DNA replication, and mismatch repair. We also identified some gene mutations (ATRX (57), and MUC16) in different stemness subtypes, which might be potential targets for STS to regulate stemness of tumor cells. Besides, high stemness was also associated with more CNA. It is noted that genomic instability including gene mutations and CNA tends to result in more immune infiltration. However, high-stemness subtypes with more gene mutations and CNA possessed low immune cells, which might be caused by more invalid antigens or lower antigen presentation. It is noted that tumor immunity is a complex biological phenomenon, and further studies are required to completely clarify inconsistency between genomic changes and immune infiltration.

Epigenetic regulation was closely associated with growth and development, maintenance of normal cell function, genome integrity, and transcriptional regulation. DNA methylation as a form of epigenetic regulation was reported to negatively regulate gene expression to drive tumor formation and maintain stemness (58, 59). We found that stemness of STS might be regulated by DNA methylation. We also identified CPXM2, CYP1B1, and DES as potential stemness-related methylation-driven genes, which might contribute to the progression of STS. CYP1B1 was reported to drive cancer cell stemness and patient outcome in head-and-neck carcinoma (52). CPXM2 was associated with poor prognosis in gastric cancer (60). DES was also reported in multiple cancers (61).

Considering the important roles of stemness in the prediction of prognosis and treatment, we developed a quantitative tool, SPi, to contribute to the clinical application of stemness in STS. In this study, clinical and genomic characteristics of SPi were described as follows: 1) Compared with mRNAsi, SPi had higher predictive value of prognosis in STS. As expected, high SPi was associated with high stemness, low infiltration of immune cells, poor prognosis, and other genomic characteristics similar to stemness subtypes. 2) SPi also had important clinical characteristics. High SPi was observed in patients aged >65, diagnosed with UPS and metastases, consistent with previous studies where age and presence of metastases were reported as risk factors in STS (62, 63). 3) In addition to the high predictive value of prognosis, SPi was expected to possess guide significance for immunotherapy. According to characteristics of the immune microenvironment and the expression of immune checkpoints, we speculated that SPi could predict the efficacy of patients treated with immunotherapy. As we expected and speculated, patients with low SPi and low-stemness subtype were more likely to respond to immunotherapy. 4) Considering the close relationships of MSI with SPi, we investigated whether the combination of MSI and SPi could make up shortcomings of each other and improve the prediction of prognosis and management. We found that the combination of MSI and SPi contributed to elevated efficacy of SPi to predict the prognosis and benefits of patients from immunotherapy. Patients who meet both low MSI and SPi were more likely to respond to immunotherapy.

To our knowledge, it is the first study to comprehensively reveal the role of stemness in STS based on muti-omic data. Based on the above findings, our study had several significances and clinical applications. We identified three stemness subtypes with distinct stemness, immune, and metabolic characteristics, which contribute to understanding of relationships among stemness, immunity, and metabolism. The low response rate is the main difficulty of immunotherapy, especially in STS (64). Most tumors were considered as “cold tumors” insensitive to immunotherapy. Knowledge of stemness could also be exploited to remodel the immune microenvironment and tumor metabolism to benefit “cold tumors” from immunotherapy. We also developed an SRS-based tool (SPi) to promote the clinical application of stemness, and SPi could predict the prognosis and benefits of patients from immunotherapy. However, our study had the inevitable disadvantage of small sample size (568 patients). Due to the lower incidence of STS, we considered this sample size to be acceptable. Furthermore, muti-omic data and multiple methods could make up for the disadvantage of small sample size and guarantee the accuracy of this study.

## Conclusion

We identified three stemness subtypes with distinct stemness, immune, and metabolic characteristics, and developed SPi to well predict prognosis and response of patients to immunotherapy. Besides, MSI could help SPi to improve the predictive value of SPi in STS. Patients who meet both low MSI and SPi were more likely to respond to immunotherapy. Based on muti-omic analysis, this study could contribute to better understand stemness, immune, and metabolic characteristics in STS and promote the management of STS. Our study also provides a reference for study of stemness in other tumors.

## Data Availability Statement

All data used in this work can be acquired from the Gene Expression Omnibus (GEO; https://www.ncbi.nlm.nih.gov/geo/) under the accession number GSE21050, the GDC portal (https://portal.gdc.cancer.gov/), and the UCSC Xena browser (https://gdc.xenahubs.net).

## Ethics Statement

The patient data in this work were acquired from the publicly available datasets whose informed consent of patients were complete.

## Author Contributions

H-YG, A-XY, and B-WQ conceived and designed this study. H-YG and H-HP carried out the analysis procedure. W-QQ and Y-FY analyzed the results. H-YG and W-QQ contributed the analysis tools. H-YG, W-QQ, Z-ZJ, and A-XY participated in the manuscript writing. All authors contributed to the article and approved the submitted version.

## Funding

This work was supported by the Health Commission of Hubei Province Medical Leading Talent Project (Grant No. LJ20200405).

## Conflict of Interest

The authors declare that the research was conducted in the absence of any commercial or financial relationships that could be construed as a potential conflict of interest.

## Publisher’s Note

All claims expressed in this article are solely those of the authors and do not necessarily represent those of their affiliated organizations, or those of the publisher, the editors and the reviewers. Any product that may be evaluated in this article, or claim that may be made by its manufacturer, is not guaranteed or endorsed by the publisher.

## Supplementary Material

Supplementary Table 1 Identification of prognostic SRSs using univariate Cox regression analysis.

Supplementary Table 2 Identification of DEGs from different stemness subtypes.

Supplementary Table 3 Identification of differential DNA methylation sites between patients with highest (Cluster A) and lowest (Cluster C) stemness.

Supplementary Figure 1 Identification of three stemness subtypes based on prognostic SRSs in TCGA-SARC cohort. Note: Consensus clustering (K-means) algorithm was performed for TCGA-SARC patients. **(A)** CDF plot. The flatter the middle part of the curve, the better the clustering effect. **(B)** Consensus matrix plots. **(C)** Heatmap of gene expression of prognostic SRSs among three stemness subtypes (Cluster A, B and C). **(D)** K-M survival analysis in Cluster A, B and C. **(E)** The percentage of metastatic and non-metastatic patients. Na: data was not available.

Supplementary Figure 2 Identification of three stemness subtypes based on prognostic SRSs in GSE21050 cohort. Note: Consensus clustering (K-means) algorithm was performed for GSE21050 patients. **(A)** CDF plot. The flatter the middle part of the curve, the better the clustering effect. **(B)** Consensus matrix plots. **(C)** Heatmap of gene expression of prognostic SRSs among three stemness subtypes (Cluster A, B and C). **(D)** K-M survival analysis in Cluster A, B and C. **(E)** The percentage of metastatic and non-metastatic patients. Na: data was not available.

Supplementary Figure 3 Landscape of immune infiltration among three subtypes in TCGA-SARC cohort.Note: The differences of immune **(A)** and stromal **(B)** scores among three subtypes (Cluster A, B and C). **(C)** Heatmap of immune or stromal cells (calculated by xCell method) among three subtypes. **(D)** The differences of immune or stromal cells (calculated by CIBERSORT method) among three subtypes. **(E)** The differences of enrichment scores of 29 immune gene sets reflective of innate and adaptive immunity among three subtypes. ****p < 0.0001; ***p < 0.001; **0.001 < p < 0.01; *0.01 < p < 0.05; ns (not significant), p > 0.05.

Supplementary Figure 4 Landscape of immune infiltration among three subtypes in GSE21050 cohort.Note: The differences of immune **(A)** and stromal **(B)** scores among three subtypes (Cluster A, B and C). **(C)** Heatmap of immune or stromal cells (calculated by xCell method) among three subtypes. **(D)** The differences of immune or stromal cells (calculated by CIBERSORT method) among three subtypes. **(E)** The differences of enrichment scores of 29 immune gene sets reflective of innate and adaptive immunity among three subtypes. ****p < 0.0001; ***p < 0.001; **0.001 < p < 0.01; *0.01 < p < 0.05; ns (not significant), p > 0.05.

Supplementary Figure 5 The prognostic role and clinical characteristics of SPi. Note: K-M survival analyses of patients with high and low SPi in TCGA-SARC **(A)** and GSE21050 **(B)** cohorts. **(C)** The differences of SPi in patients with different clinical characteristics in TCGA-SARC cohorts.

Supplementary Figure 6 The differences of tumor immunogenicity indicators between patients with high and low SPi.

Supplementary Figure 7 Patients with low stemness could benefit from immunotherapy.Note: The differences of TIS and TIDE among three stemness subtypes in overall cohort **(A, B)**, TCGA-SARC cohort **(C, D)** GSE21050 cohort **(E, F)**. **(G)** The response of patients (Cluster A, Cluster B and Cluster C) to PD1 and CTLA4 inhibitors (Benjamini and Hochberg corrected p<0.05).
