# Supplementary figures and images for: Stemness Subtypes and Scoring System Predict Prognosis and Efficacy of Immunotherapy in Soft Tissue Sarcoma
Source: Front Immunol. 2022 Apr 7;13:796606. doi: 10.3389/fimmu.2022.796606 (PMC9022121; doi:10.3389/fimmu.2022.796606)

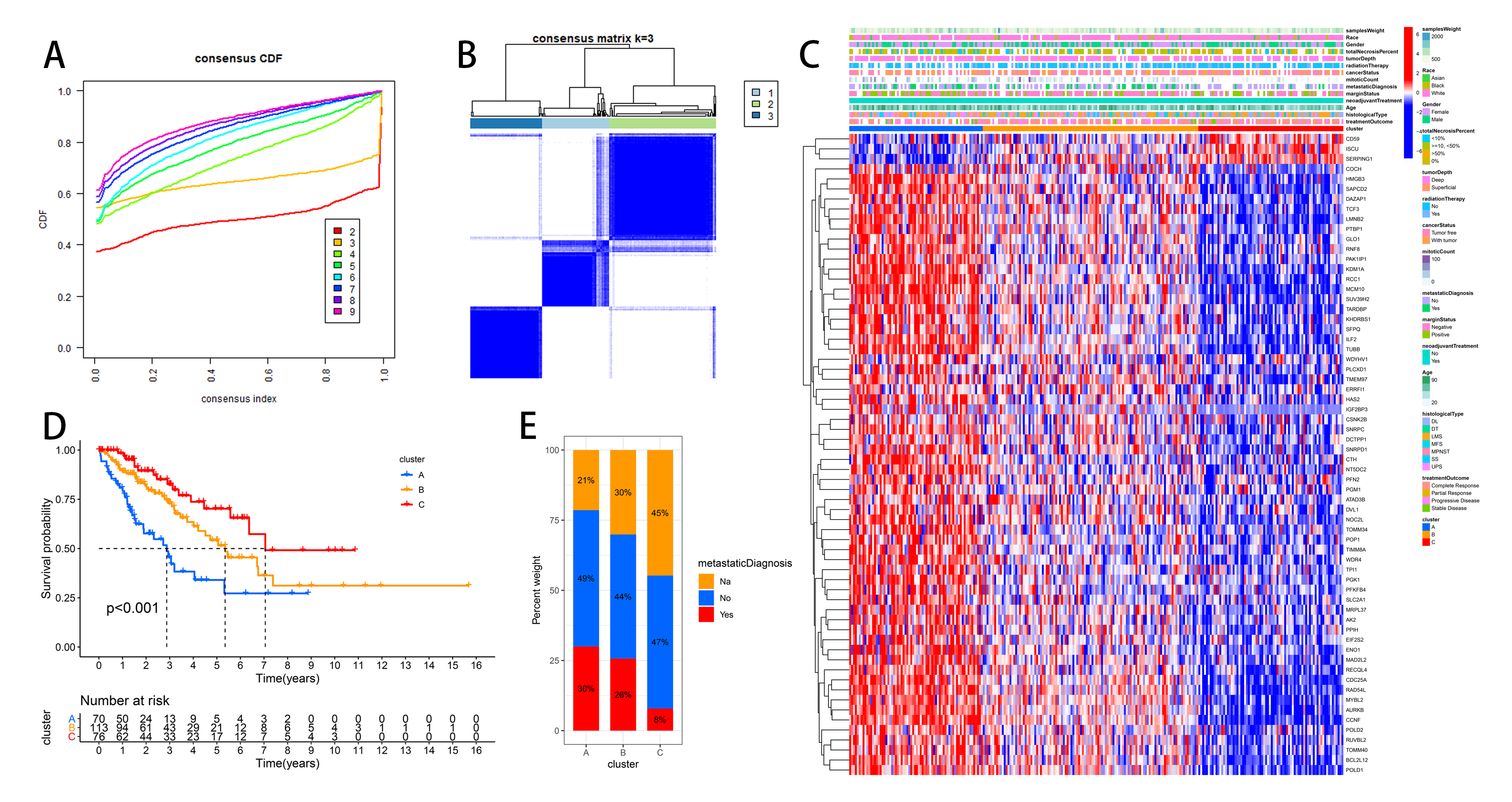

Supplement: Supplementary Figure 1 — Identification of three stemness subtypes based on prognostic SRSs in TCGA-SARC cohort. Note: Consensus clustering (K-means) algorithm was performed for TCGA-SARC patients. (A) CDF plot. The flatter the middle part of the curve, the better the clustering effect. (B) Consensus matrix plots. (C) Heatmap of gene expression of prognostic SRSs among three stemness subtypes (Cluster A, B and C). (D) K-M survival analysis in Cluster A, B and C. (E) The percentage of metastatic and non-metastatic patients. Na: data was not available. [file Image_1.tif]

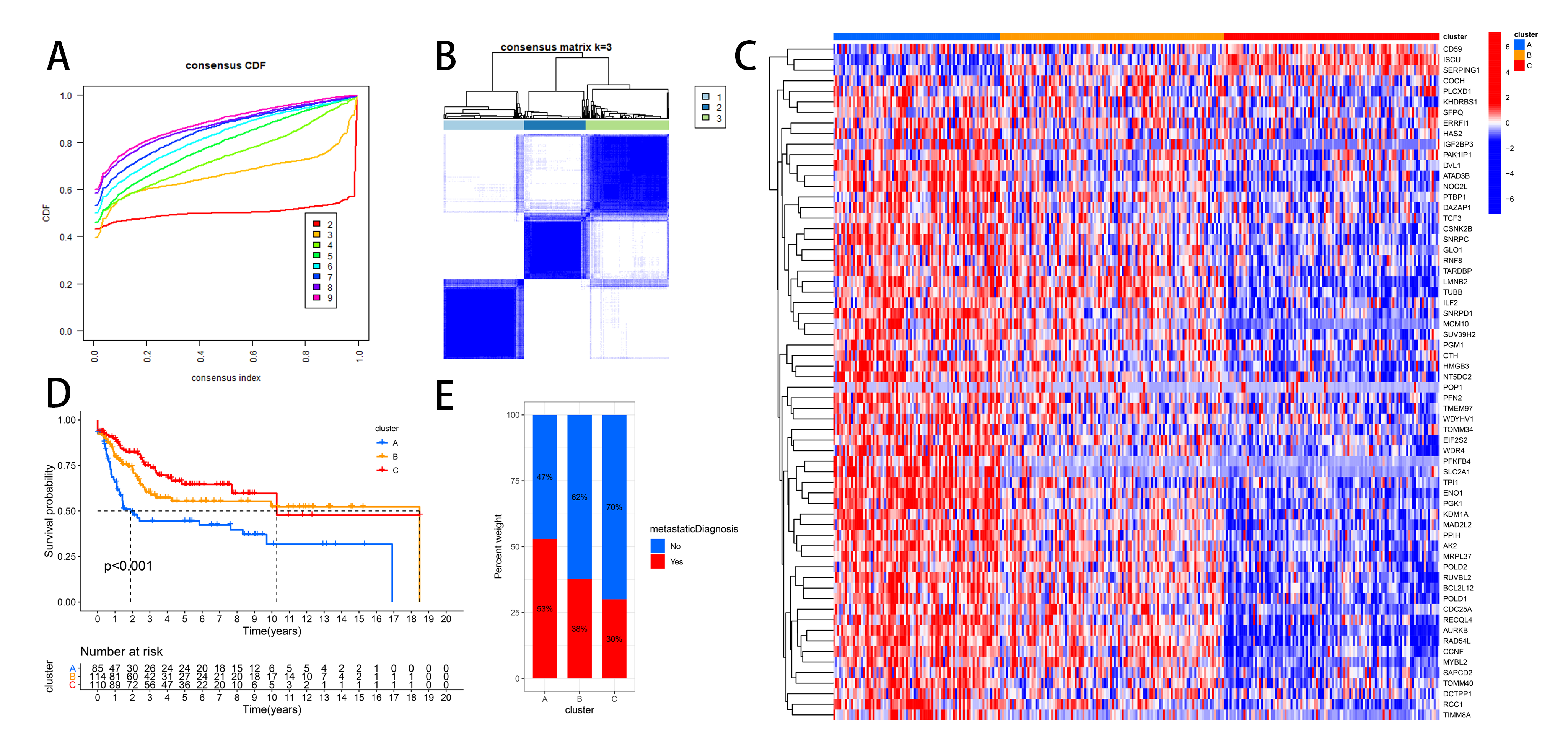

Supplement: Supplementary Figure 2 — Identification of three stemness subtypes based on prognostic SRSs in GSE21050 cohort. Note: Consensus clustering (K-means) algorithm was performed for GSE21050 patients. (A) CDF plot. The flatter the middle part of the curve, the better the clustering effect. (B) Consensus matrix plots. (C) Heatmap of gene expression of prognostic SRSs among three stemness subtypes (Cluster A, B and C). (D) K-M survival analysis in Cluster A, B and C. (E) The percentage of metastatic and non-metastatic patients. Na: data was not available. [file Image_2.tif]

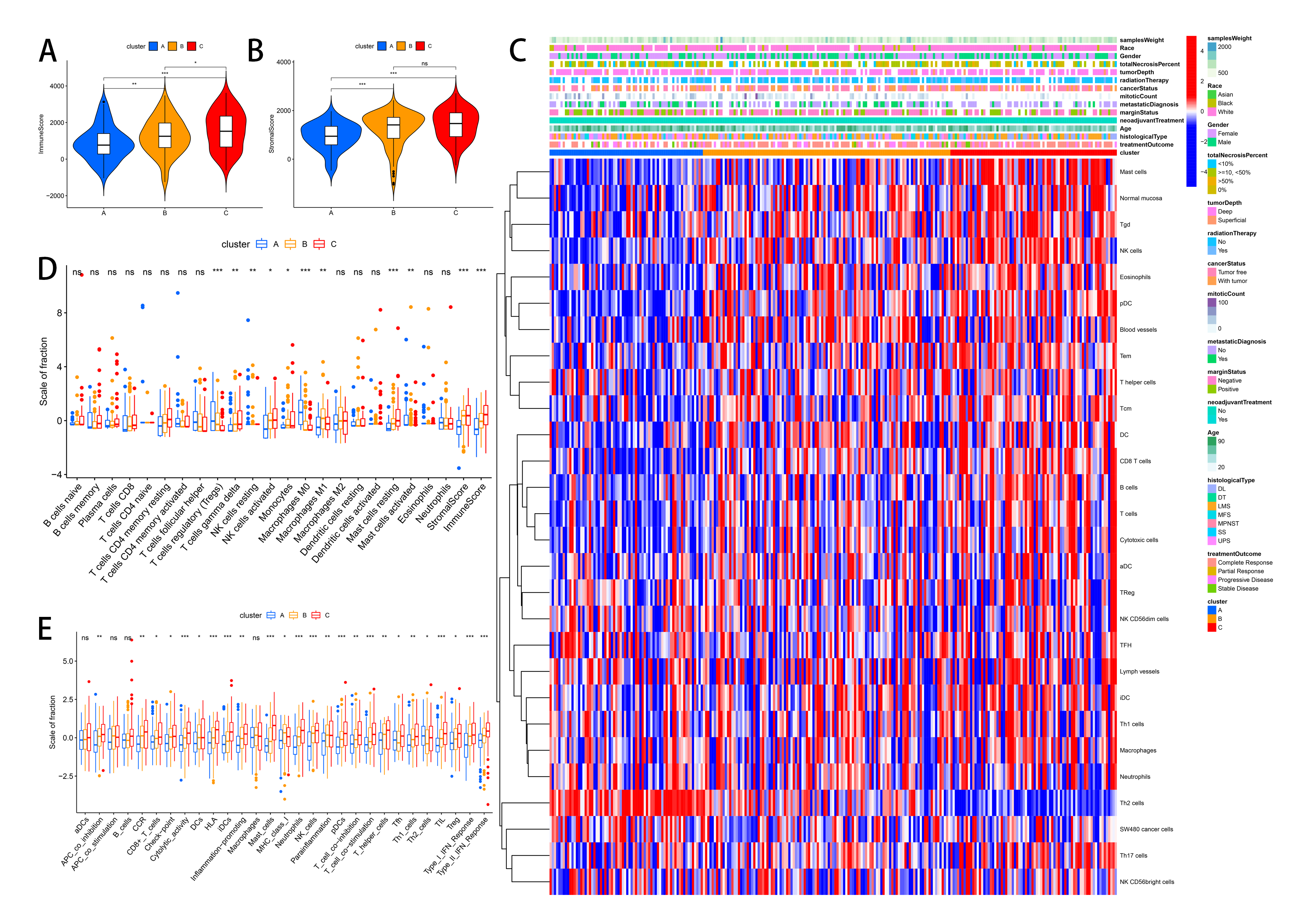

Supplement: Supplementary Figure 3 — Landscape of immune infiltration among three subtypes in TCGA-SARC cohort.Note: The differences of immune (A) and stromal (B) scores among three subtypes (Cluster A, B and C). (C) Heatmap of immune or stromal cells (calculated by xCell method) among three subtypes. (D) The differences of immune or stromal cells (calculated by CIBERSORT method) among three subtypes. (E) The differences of enrichment scores of 29 immune gene sets reflective of innate and adaptive immunity among three subtypes. ****p < 0.0001; ***p < 0.001; **0.001 < p < 0.01; *0.01 < p < 0.05; ns (not significant), p > 0.05. [file Image_3.tif]

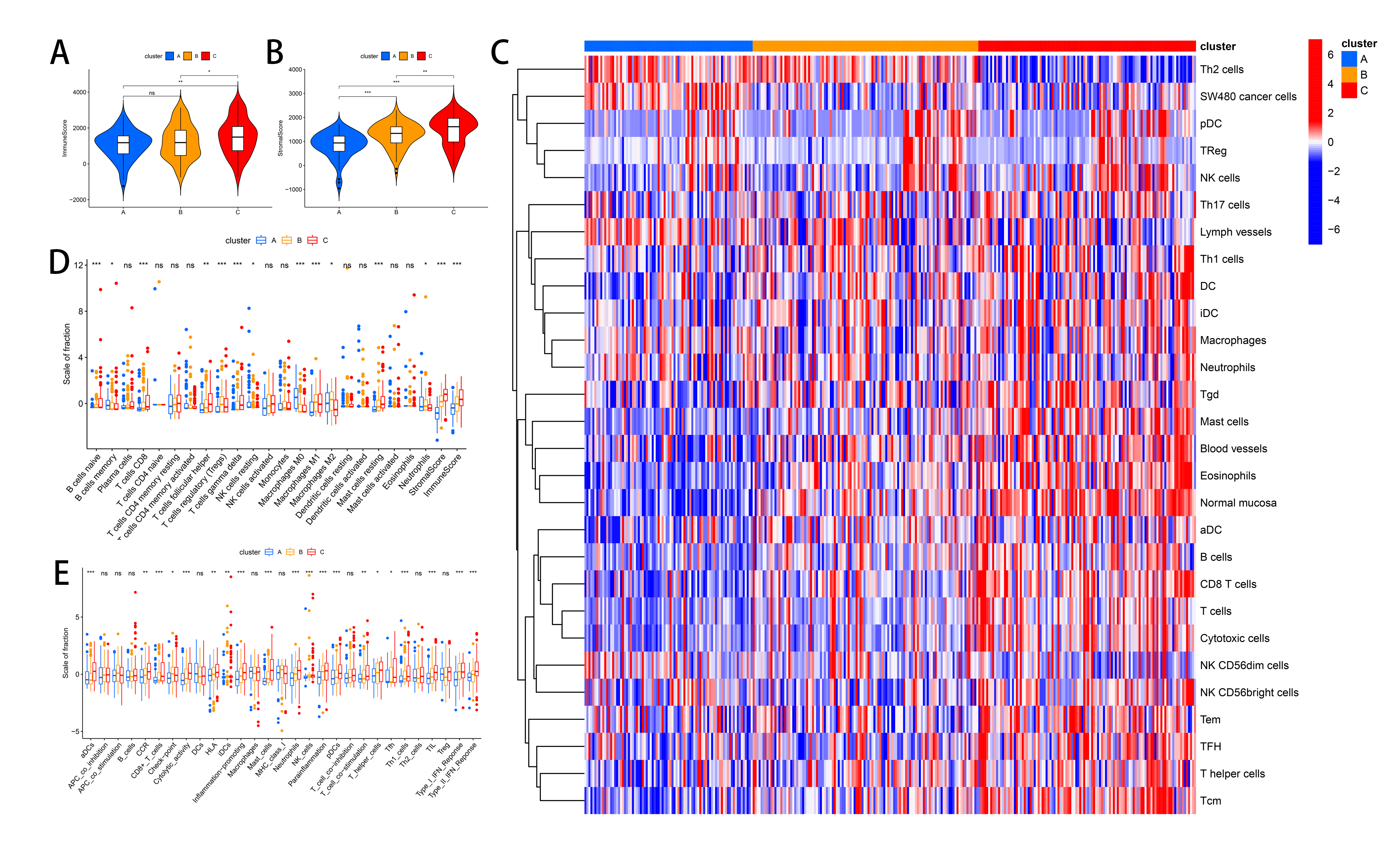

Supplement: Supplementary Figure 4 — Landscape of immune infiltration among three subtypes in GSE21050 cohort.Note: The differences of immune (A) and stromal (B) scores among three subtypes (Cluster A, B and C). (C) Heatmap of immune or stromal cells (calculated by xCell method) among three subtypes. (D) The differences of immune or stromal cells (calculated by CIBERSORT method) among three subtypes. (E) The differences of enrichment scores of 29 immune gene sets reflective of innate and adaptive immunity among three subtypes. ****p < 0.0001; ***p < 0.001; **0.001 < p < 0.01; *0.01 < p < 0.05; ns (not significant), p > 0.05. [file Image_4.tif]

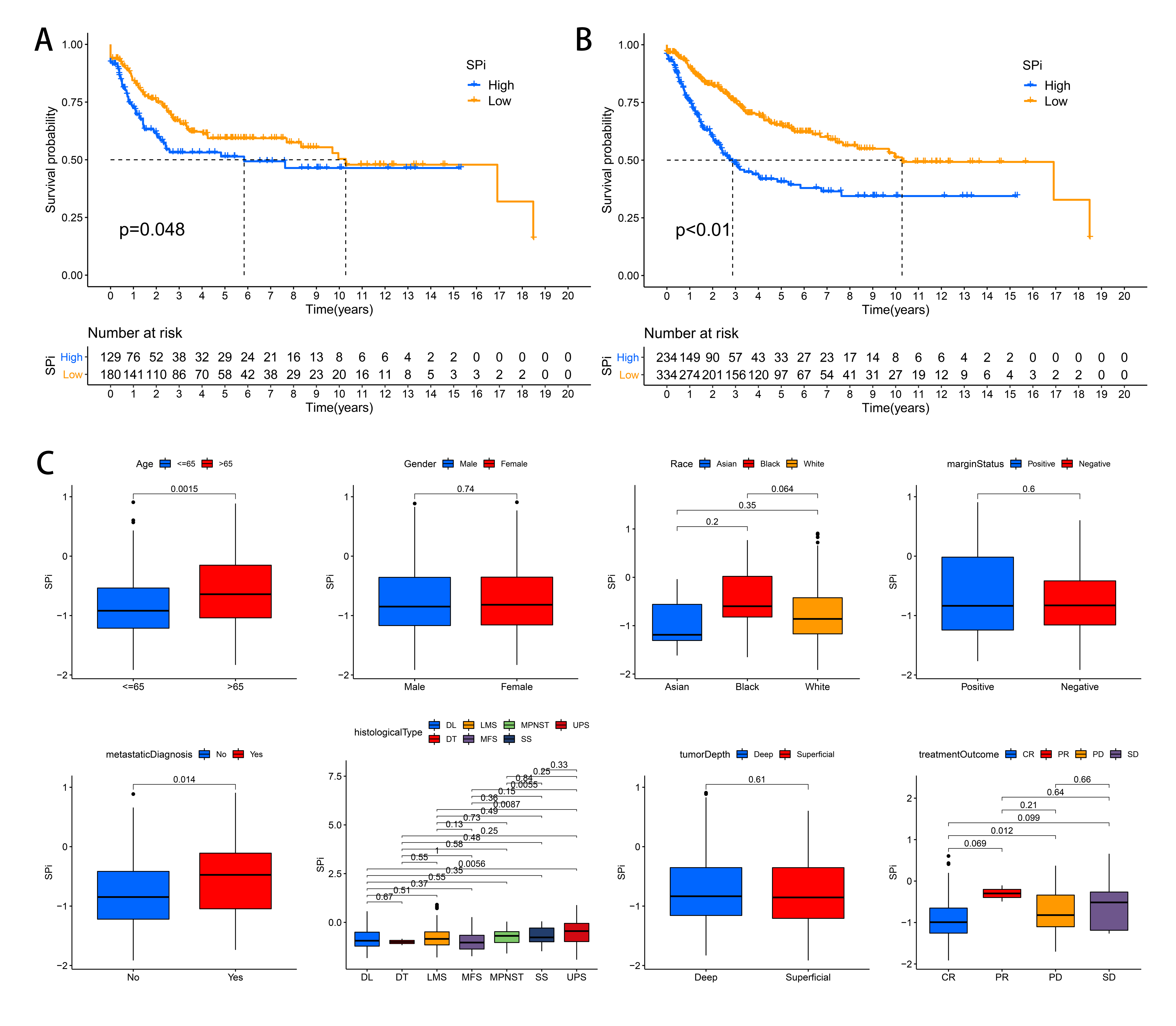

Supplement: Supplementary Figure 5 — The prognostic role and clinical characteristics of SPi. Note: K-M survival analyses of patients with high and low SPi in TCGA-SARC (A) and GSE21050 (B) cohorts. (C) The differences of SPi in patients with different clinical characteristics in TCGA-SARC cohorts. [file Image_5.tif]

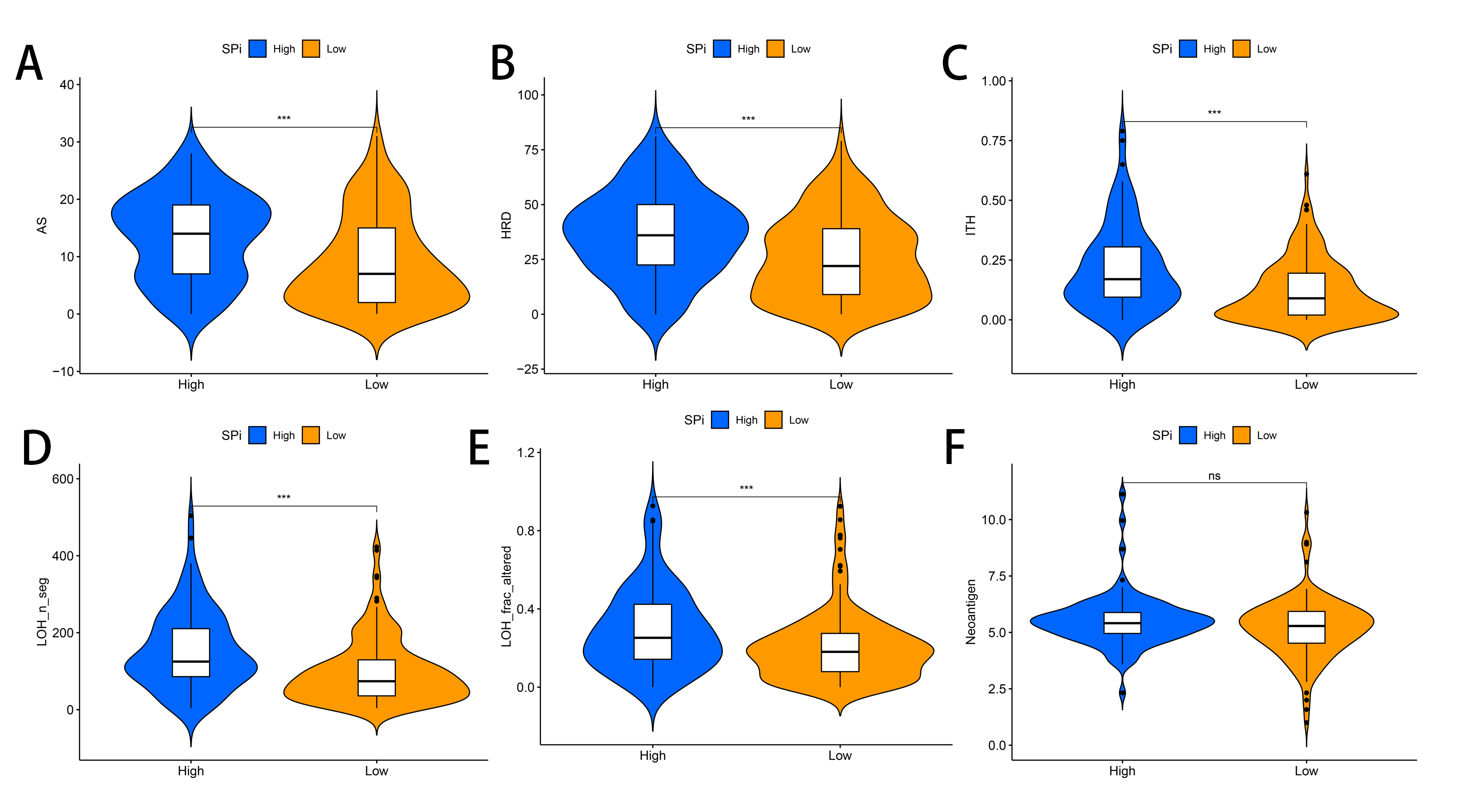

Supplement: Supplementary Figure 6 — The differences of tumor immunogenicity indicators between patients with high and low SPi. [file Image_6.tif]

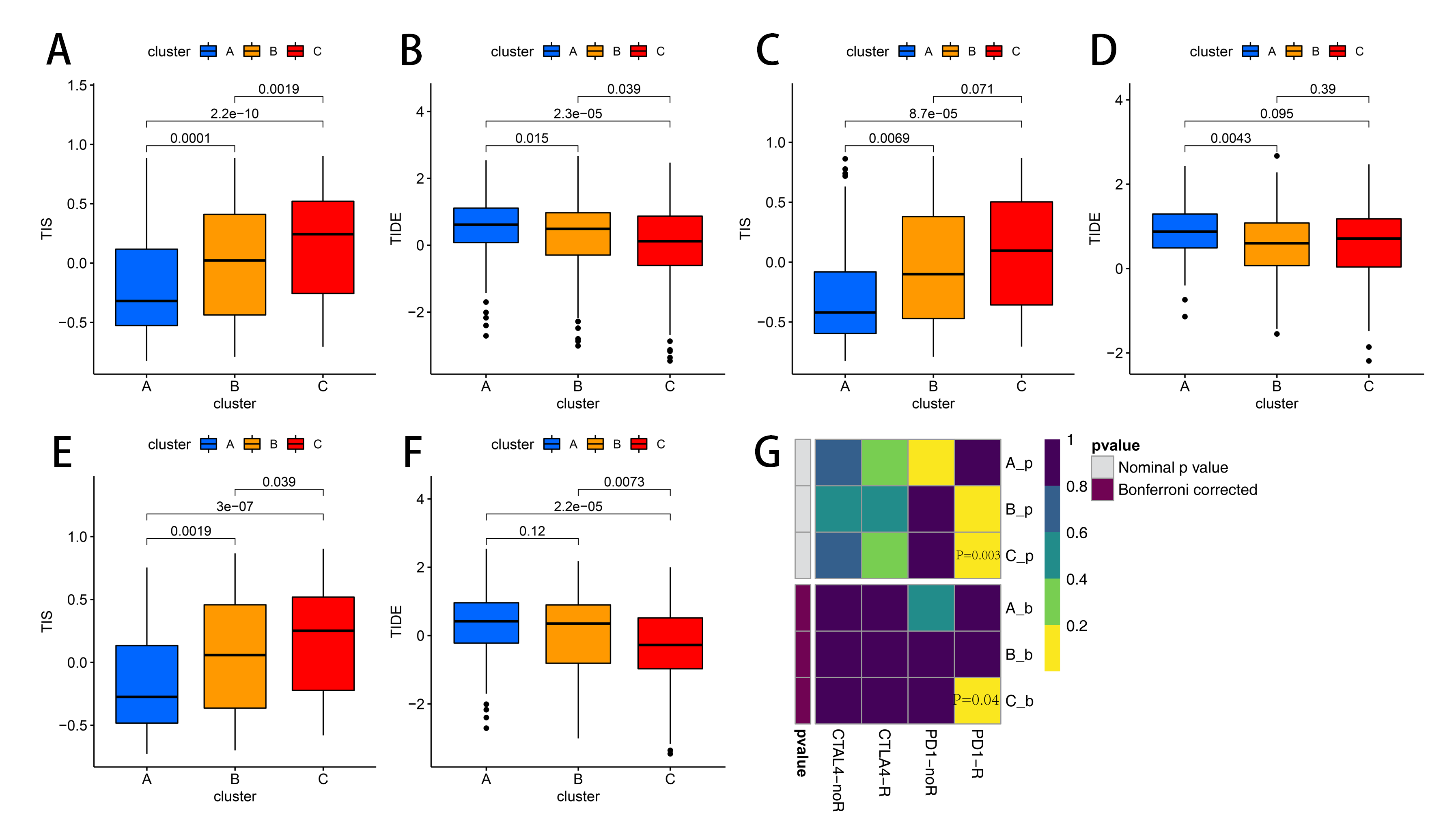

Supplement: Supplementary Figure 7 — Patients with low stemness could benefit from immunotherapy.Note: The differences of TIS and TIDE among three stemness subtypes in overall cohort (A, B), TCGA-SARC cohort (C, D) GSE21050 cohort (E, F). (G) The response of patients (Cluster A, Cluster B and Cluster C) to PD1 and CTLA4 inhibitors (Benjamini and Hochberg corrected p<0.05). [file Image_7.tif]
